# Metformin Attenuates Tau Pathology in Tau-Seeded PS19 Mice

**DOI:** 10.1007/s13311-022-01316-6

**Published:** 2022-11-23

**Authors:** Shuai Zhao, Ziqi Fan, Xinyi Zhang, Zheyu Li, Ting Shen, Kaicheng Li, Yaping Yan, Yunfeng Yuan, Jiali Pu, Jun Tian, Zhirong Liu, Yanxing Chen, Baorong Zhang

**Affiliations:** 1 Department of Neurology, The Second Affiliated Hospital, School of Medicine, Zhejiang University, Hangzhou, People’s Republic of China; 2 Department of Neurology, The First Affiliated Hospital, School of Medicine, Zhejiang University, Hangzhou, People’s Republic of China

**Keywords:** Alzheimer’s disease, Metformin, Tau pathology, Tau spreading, Tau hyperphosphorylation

## Abstract

Accumulation of neurofibrillary tangles (NFTs) composed of hyperphosphorylated tau is a histopathological hallmark of Alzheimer’s disease (AD) and related tauopathies. Growing evidence demonstrated that tau pathology in AD spreads in a prion-like manner. Previous studies showed that metformin might have a positive effect on cognition. However, the underlying mechanisms are still unknown. Therefore, the present study aimed to investigate the effects of metformin on tau propagation. Brain extracts containing tau aggregates were unilaterally injected into the hippocampus and the overlying cerebral cortex of PS19 mice. Metformin was administrated through drinking water for four months, and we observed tau spreading in the brain of tau-seeded PS19 mice. Metformin inhibited the spreading of tau pathology in the ipsilateral hemisphere, attenuated tau pathology in the contralateral hemisphere, and reduced the hyperphosphorylation of tau at Ser202/Thr205, Thr231, and Ser422 sites in the soluble fraction and Ser202/Thr205, Ser262, Thr396, Thr231, and Ser422 sites in the insoluble fraction of tau-seeded PS19 mice brains. Metformin did not affect tau kinases or phosphatase 2A protein levels but reduced mTORC1 protein levels. Additionally, metformin reduced learning and memory deficits of the tau-seeded PS19 mice. These findings indicate that metformin reduced tau hyperphosphorylation, attenuated tau pathology in tau-seeded PS19 mice, and improved learning and memory deficits. These findings highlight the potential mechanisms underlying the beneficial effects of metformin on cognition, implying that metformin could be a promising drug for the prevention and early treatment of AD.

**Supplementary Information:**

The online version contains supplementary material available at 10.1007/s13311-022-01316-6.

## Introduction

Hyperphosphorylated tau neurofibrillary tangles (NFTs) are one of the most important histopathological hallmarks of Alzheimer’s disease (AD) and other tauopathies [1]. As a microtubule-associated protein, tau binds with microtubules to maintain the structure and stability of neurons under normal circumstances. When tau protein is abnormally phosphorylated, it misfolded and aggregated to form NFTs in neurons, resulting in the loss of functional microtubules and, eventually, the death of neurons [2]. Tau pathology is closely associated with the cognitive performance of AD patients. The Braak stage, the neuropathological diagnostic criteria of AD, determines the stage of AD based on the progression of NFTs [3]. Tau pathology develops in a stereotypical pattern in selectively vulnerable brain regions, first in the entorhinal cortex, then in the hippocampus, and finally in the entire neocortex [4].

Many neurodegenerative diseases are associated with the accumulation of β-sheet-rich aggregates. These diseases share characteristics with prionopathies, such as phenotypic diversity and the pathology spread [5]. Numerous studies suggest that tau and prion proteins have similar biochemical characteristics [6]. Pathological tau can transmit between neurons in a prion-like self-propagating mechanism [7]. In mouse models, expressing P301L mutated h-Tau restricted to the entorhinal cortex (EC) resulted in tau pathology spreading from EC to connected brain regions [8]. Intracerebral injection of synthetic preformed tau fibrils or brain extracts containing tau aggregates resulted in tau pathology propagating from injected sites to neurons with synaptic connections in a dose and time-dependent manner [9–11]. Altogether, it is well established that tau pathology can spread to different brain regions in a prion-like manner.

Substantial evidence has indicated a link between AD and type 2 diabetes mellitus (T2DM). Studies have shown that T2DM patients have an increased risk of AD [12]. Diabetes complications include cognitive impairment and dementia [13, 14]. AD may even be considered a metabolic disease [15]. Metformin, one of the most widely used drugs for T2DM, has been shown to benefit age-related diseases such as cancer, cardiovascular disease, and neurodegenerative diseases, particularly AD [16]. Metformin reduced the risk of dementia in T2DM patients in longitudinal and cross-sectional analyses [17–19]. Long-term use of metformin is more beneficial for improving cognitive function in T2DM patients than other antidiabetic drugs [18, 19]. Animal studies have also confirmed the beneficial effects of metformin on cognition. In rodents, it was found that metformin can prevent spatial memory impairment in mice fed with a high-fat diet [20]. Metformin treatment for 6 weeks significantly improved long-term potentiation (LTP) and memory impairment in mice [21]. Another study revealed that metformin treatment improved learning behavior in high-fat diet-induced insulin-resistant rats [22].

Regarding tau pathology, metformin administration promoted tau protein dephosphorylation in vivo and in vitro [23, 24]. Our previous research found that metformin reduced tau aggregates in dystrophic neurites surrounding amyloid-β plaques (NP tau) in tau-seeded APP/PS1 mice [25], implying that metformin may be able to limit the propagation of tau pathologies. On the other hand, recent failure in the clinical trials for disease modifying therapies for AD suggest that the disease should be treated at an earlier stage. Importantly, preventing tau spreading at the early stage of AD may be the key to develop treatment for AD. It remains unknown whether metformin can slow or inhibit the pathological transmission of tau pathology. Therefore, the present study was designed to investigate the effect of metformin on tau spreading as well as cognitive function in tau-seeded PS19 mice and their underlying mechanisms.

## Methods

### Animals and Drug Treatment

All procedures were approved by the Institutional Animal Care and Use Committee of Zhejiang University and were conducted according to the National Institutes of Health Guide for the Care and Use of Laboratory Animals guidelines for the ethical treatment of animals. The number of animals used in the study was kept to a minimum. Breeding pairs of PS19 mice were obtained from the Jackson Laboratory (B6; C3-Tg (Prnp-MAPT*P301S) PS19Vle/J; stock number 008169). Mice were housed in filtered cages in a temperature-controlled room with a 12-h light/dark cycle with ad libitum food and water access.

In this study, only male mice were used. PS19 mice and wild-type (WT) mice were randomly assigned to the treatment or vehicle group at 2.5 months of age. Mice in the treatment group were given 4 mg/mL metformin (Sigma, St. Louis, MO, USA) in their drinking water for four months. In contrast, the mice in the vehicle group were given normal drinking water. Every week, drinking bottles were replenished with fresh water or metformin solution. Mice were sacrificed after four months of metformin treatment. Supplementary Fig. 1 illustrates the complete experiment design.

### Preparation of Brain Extracts and Infusion Procedure

Brain extracts were prepared from end-stage PS19 mice (^PS19^BE) and age-matched wild-type control mice (^WT^BE) by the previously described procedure [9]. Briefly, mice were sacrificed, and their cerebral cortices and hippocampi were frozen in dry ice. Brain tissue was homogenized in sterile phosphate-buffered saline (PBS) at 10% (w/v), sonicated and centrifuged at 3000 g for 5 min at 4 °C. The supernatant was stored at −80 °C until it was used. The brain extracts were used at a final concentration of 6.38 mg/mL total protein, as measured by the bicinchoninic acid (BCA) protein assay kit. A dot blot was performed to measure the tau protein concentration. A standard curve was prepared using recombinant tau. The tau concentration in brain extracts was calculated as 61.82 µg/mL (Supplementary Fig. 2a). The brain extracts were characterized using negative staining transmission electron microscopy, which revealed straight filaments (Supplementary Fig. 2b). Mice were deeply anesthetized with Avertin (Sigma, St. Louis, MO, USA). The intracerebral infusions were conducted according to our previously described method [25]. Briefly, mice were placed in a stereotaxic frame, and a heating blanket was used to maintain the body temperature at 38 ± 1 °C. The skull was exposed, cleaned, and a hole was drilled. A Hamilton syringe unilaterally injected brain extracts.

At a flow rate of 1.25 µL/min, 2.5 µL inoculum was injected into the hippocampus (bregma, −2.50 mm; lateral, +2.00 mm; and depth, −1.80 mm) and the overlying cortex (bregma, −2.50 mm; lateral, +2.00 mm; and depth, −0.80 mm). The needle was kept in place for an additional 3 min before being removed slowly. Mice were returned to their cages after fully recovering on the heating pad.

### Immunofluorescence

Four months after treatment, mice were deeply anesthetized and transcardially perfused with cold PBS, followed by 4% paraformaldehyde. Mouse brains were collected, fixed overnight in buffered 4% paraformaldehyde at 4 °C, and dehydrated in buffered 30% sucrose solution. The brains were then cut into 30-μm serial coronal sections on a cryostat, and the free-floating sections were stored at − 20 °C in an anti-freeze solution (glycerol, ethylene glycol in 0.1 M PBS) till further use.

Immunofluorescence was performed as described previously [25]. Brain sections were permeabilized with 0.3% Triton X-100 and blocked with 5% normal goat serum for 30 min before incubating with primary antibodies overnight at 4 °C. Normal goat serum without primary antibodies was used as a negative control. The next morning, sections were incubated with the appropriate secondary antibody for 1 h at room temperature in the dark. The sections were then incubated with 4,6-diamidino-2-phenylindole (DAPI) for 5 min. Samples were washed with PBS and mounted on microscope slides with SlowFade Gold antifade reagent.

### Western Blot

Mice were deeply anesthetized before being transcardially perfused with cold PBS. Mouse brains were immediately removed, dissecting different brain regions and snap-frozen separately in dry ice. Frozen brain tissues were homogenized in ice-cold buffer (50 mM Tris–HCl (pH 7.4), 2.0 mM EGTA, 2 mM Na_3_VO_4_, 50 mM NaF, 20 mM β-glycerophosphate, 0.5 mM AEBSF, 10 μg/mL aprotinin, 10 μg/mL leupeptin, and 4 μg/mL pepstatin A), containing protease inhibitor cocktail, phosphatase inhibitor and phenylmethyl sulfonyl fluoride (PMSF). The sarkosyl insoluble fraction was prepared by the previously described method [26]. Briefly, brain tissue was homogenized with cold high-salt RAB buffer (0.75 M NaCl, 0.02 M NaF, 100 mM Tris, 2 mM DTT, 1 mM EGTA, 0.5 mM MgSO_4_, containing protease inhibitor cocktail, phosphatase inhibitor and PMSF), then sonicated and centrifuged at 100,000 g for 30 min at 4 °C. The pellets were resonicated in high-salt RAB buffer containing 1% Triton X-100 before being centrifuged at 100,000 g for 30 min. The pellets were resonicated in PHF buffer (10 mM Tris, 0.8 M NaCl, 1 mM EDTA, 2 mM DTT, 10% sucrose, containing 1% sarkosyl, protease inhibitor cocktail, phosphatase inhibitor and PMSF), rotated for 1 h at room temperature, and then centrifuged again at 100,000 g for 30 min at 4 °C. The pellets were washed and resuspended in PBS and sonicated. The resulting homogenates were collected as the sarkosyl insoluble fraction.

Proteins were separated by gel electrophoresis and transferred to a poly(vinylidene fluoride) (PVDF) membrane (Millipore, Bedford, MA, USA) for Western blot. The membranes were blocked in 5% skimmed milk in 0.1% tris-buffered saline/Tween-20 (TBST) for 1 h after transfer and then incubated overnight at 4 °C with primary antibodies. The next day, the membranes were incubated for 1 h at room temperature with the appropriate secondary antibody. The ECL-PLUS system then visualized positive antibody binding with enhanced chemiluminescence (ECL, Pierce, Rockford, IL, USA). Signal intensity was then analyzed with Image Lab (BIO-RAD, Hercules, CA, USA).

### Morris Water Maze

The Morris water maze (MWM) task was performed after four months of metformin or vehicle treatment. A circular pool with a diameter of 120 cm and a height of 50 cm was filled with water. The water temperature was kept constant at 22 ± 1 °C. Imaging lines conceptually divided the pool into four equal quadrants. A 10-cm-diameter platform was placed in the center of the target quadrant and submerged 1.0 cm below the surface of the water. The mice were trained to find the platform and subjected to four daily trials for three consecutive days for spatial acquisition. If the mouse did not find the hidden platform within 60 s, it was manually guided to it. The mice were allowed to stay on the platform for 15 s. The probe trial was performed 24 h after the last acquisition trial. The hidden platform was removed, and mice were free to swim for 60 s. A tracking system was used to record the time to reach the platform (escape latency), time spent in the target quadrant, number of platform crossings, and swimming speed.

### Antibodies and Reagents

Table 1 lists the primary antibodies used in the present study. Horseradish peroxide (HRP)–conjugated anti-mouse and anti-rabbit secondary antibodies for Western blot were obtained from Invitrogen (Carlsbad, CA, USA). Goat anti-rabbit secondary antibody and goat anti-mouse secondary antibody for immunofluorescence were purchased from Life Technologies (Darmstadt, Germany). Other chemicals were purchased from Sigma (St. Louis, MO, USA).

**Table 1:** The primary antibodies used in this study

**Antibody**	**Source**
AT8 (pS202/pT205)	Thermo Fisher Scientific, MA, USA
p-Tau (Ser262)	Thermo Fisher Scientific, MA, USA
p-Tau (Ser396)	Thermo Fisher Scientific, MA, USA
p-Tau (Thr231)	Thermo Fisher Scientific, MA, USA
p-Tau (Ser422)	Thermo Fisher Scientific, MA, USA
tau5	Millipore, MA, USA
GAPDH	Cell Signaling Technology, MA, USA
GSK-3α/β (pS21/pS9)	Cell Signaling Technology, MA, USA
GSK-3α/β	Cell Signaling Technology, MA, USA
CDK5	Santa Cruz Biotechnology, CA, USA
P35	Cell Signaling Technology, MA, USA
ERKl/2 (pT202/pY204)	Cell Signaling Technology, MA, USA
ERKl/2	Cell Signaling Technology, MA, USA
JNK (pT183/pY185)	Cell Signaling Technology, MA, USA
JNK	Cell Signaling Technology, MA, USA
CaMKII (pT286)	Santa Cruz Biotechnology, CA, USA
CaMKII	Santa Cruz Biotechnology, CA, USA
PP2A(pY307)	Santa Cruz Biotechnology, CA, USA
PP2A-C	Millipore, MA, USA
mTORC1	Cell Signaling Technology, MA, USA
β-actin	Hua’an, Hangzhou, China

### Statistical Analysis

GraphPad Prism software (GraphPad Software Inc., San Diego, CA, USA) was used for statistical analysis. A two-way repeated-measures analysis of variance (ANOVA) was used to compare escape latency during MWM, followed by Fisher's LSD post hoc tests. A one-way ANOVA with the Bonferroni post hoc was used for other group comparisons. The Student *t*-test was used to compare the results of the two groups. Data are presented as mean ± standard error of the mean (SEM), with *P* values < 0.05 considered statistically significant.

## Results

### Injection of Proteopathic Tau Seeds Induced Tau Spreading in PS19 Mice

**Fig. 1:**
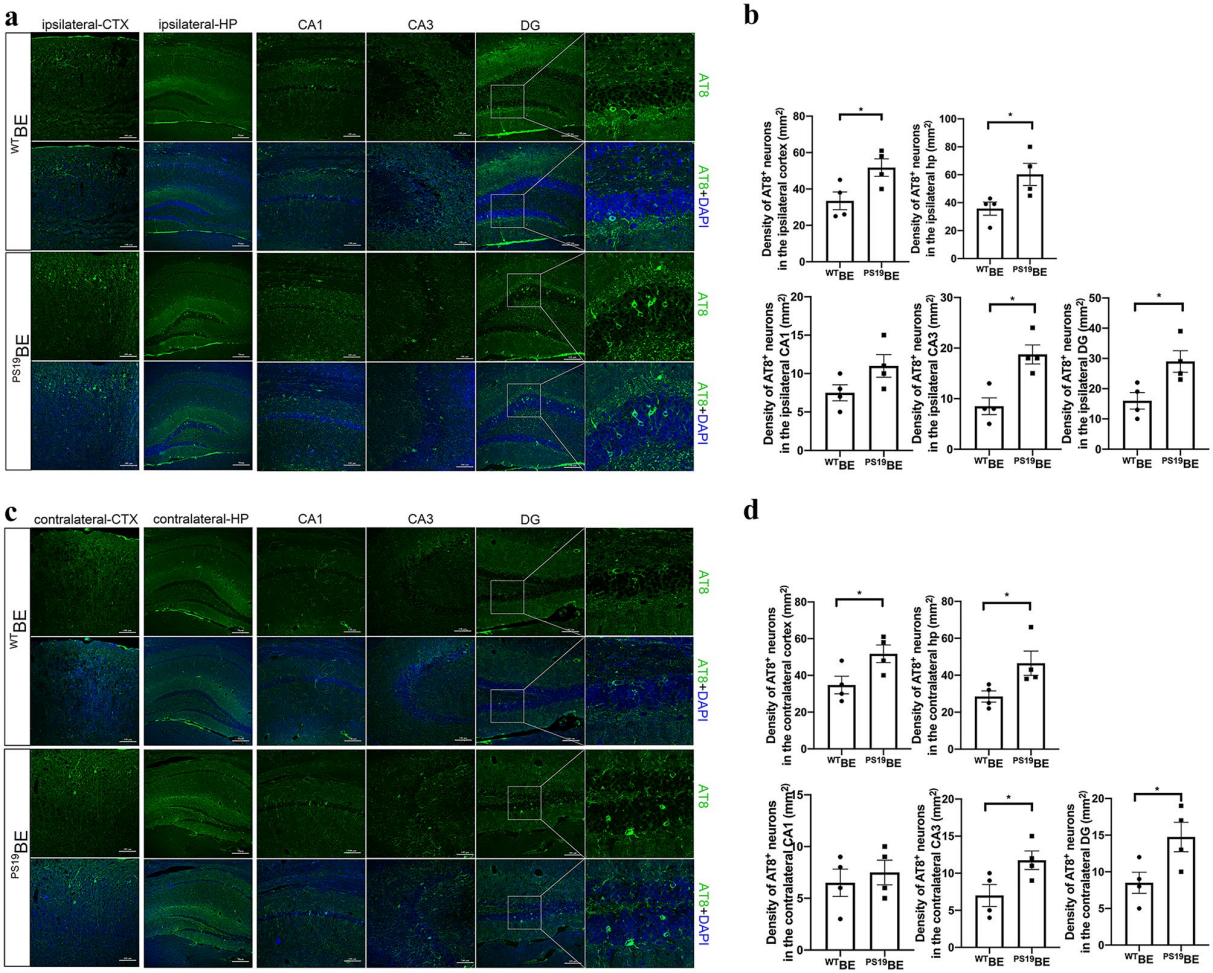
Injection of proteopathic tau seeds induced tau spreading in PS19 mice. Representative images of AT8-positive neurons in the ipsilateral (**a**) and contralateral cortices (**c**) and hippocampi of PS19 mice injected with ^PS19^BE or ^WT^BE. Quantification of AT8-positive neurons in the ipsilateral (**b**) and contralateral (**d**) cortices and hippocampi of PS19 mice. The data were analyzed by Student’s *t*-test. Values are presented as mean ± SEM. *n* = 4 per group. *P < 0.05 vs. ^WT^BE-injected PS19 mice. CTX, cortex; HP, hippocampus; DG, dentate gyrus

At 2.5 months of age, PS19 mice were unilaterally injected with ^PS19^BE or ^WT^BE. Four months after injection, we observed tau pathology spreading in the PS19 mice injected with ^PS19^BE (Fig. 1). The density of AT8-positive neurons in the bilateral cortex and hippocampus of the PS19 mice injected with ^PS19^BE was significantly higher than in PS19 mice injected with ^WT^BE. Increased AT8 staining was observed in the cortex, dentate gyrus, and CA3 subfields of the hippocampus in the ipsilateral hemisphere. In addition, an increased density of AT8-positive neurons was also observed in the contralateral cortex and hippocampus of the ^PS19^BE-injected PS19 mice (Fig. 1c, d), indicating the spreading of tau pathology to the contralateral cerebral hemisphere. These findings suggest injecting proteopathic tau seeds can cause tau spreading in PS19 mice.

### Metformin Limited Tau Pathology in Tau-Seeded PS19 Mice

**Fig. 2:**
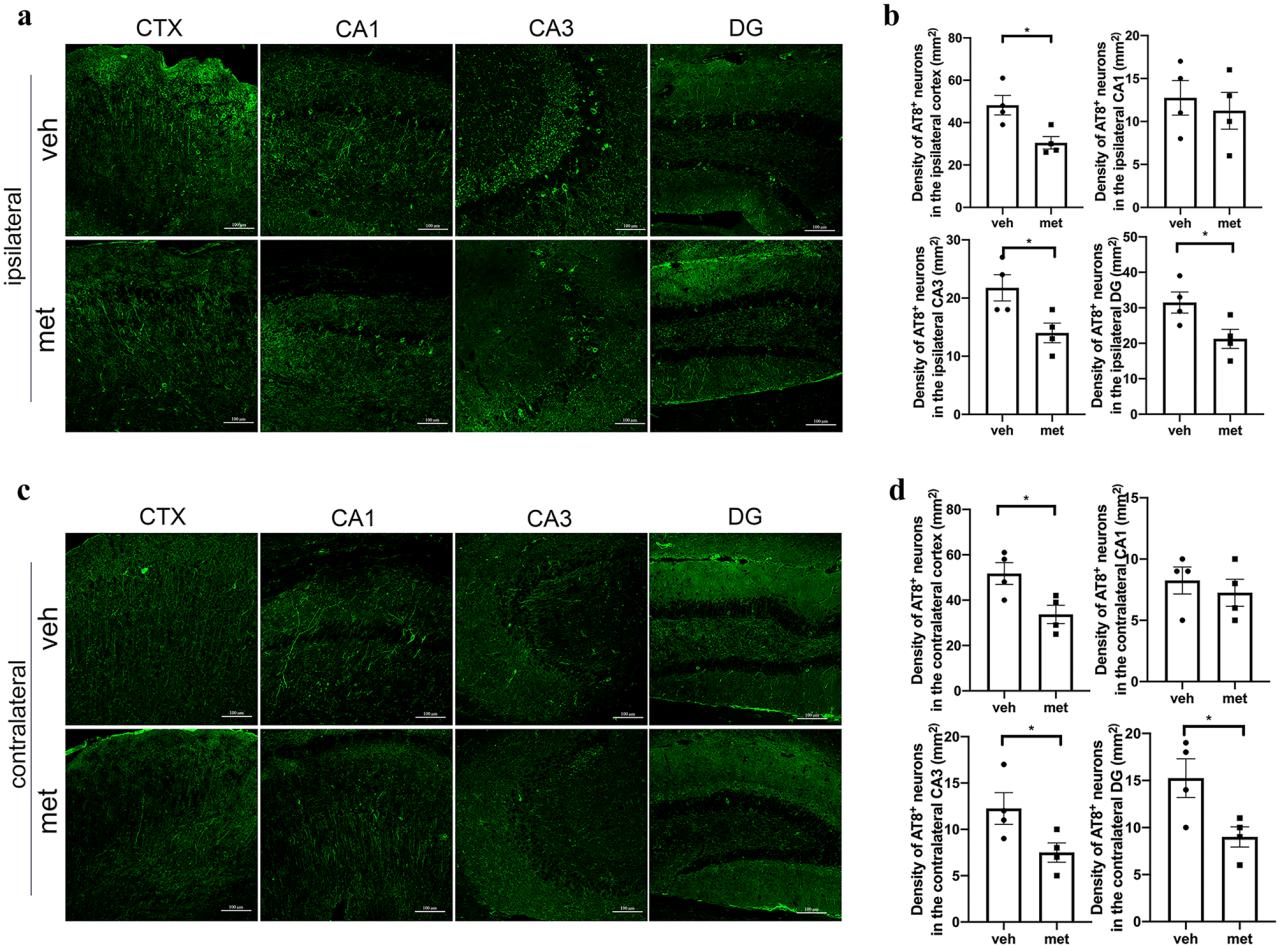
Metformin limited tau spreading in PS19 mice. Representative images of AT8-positive neurons in the ipsilateral (**a**) and contralateral (**c**) cortices and CA1, CA3, and DG subfields of the hippocampi of tau-seeded PS19 mice treated with metformin or normal drinking water. Quantification of AT8-positive neurons in the ipsilateral (**b**) and contralateral (**d**) cortices and hippocampi of PS19 mice. The data were analyzed by Student’s *t*-test. Values are presented as mean ± SEM. *n* = 4 per group. *P < 0.05 vs. tau-seeded PS19 mice treated with normal drinking water. CTX, cortex; DG, dentate gyrus; veh, vehicle; met, metformin

Tau-seeded PS19 mice were treated with metformin for 4 months to investigate the effect of metformin on tau spreading. We found a lower density of AT8-positive neurons in the bilateral cortex of metformin-treated mice compared to vehicle-treated mice (Fig. 2), implying that metformin limited tau pathology in tau-seeded PS19 mice. Furthermore, metformin significantly reduced the density of AT8-positive neurons in the bilateral dentate gyrus and CA3 subfields. However, no significant differences in the density of AT8-positive neurons were found in the bilateral CA1 subfields of the PS19 mice (Fig. 2). These results suggest that metformin limited the transmission of tau pathology in the ipsilateral hemisphere and reduced tau pathology in the contralateral hemisphere of tau-seeded PS19 mice.

### Metformin Reduced the Phosphorylation of Tau in Tau-Seeded PS19 Mice

**Fig. 3:**
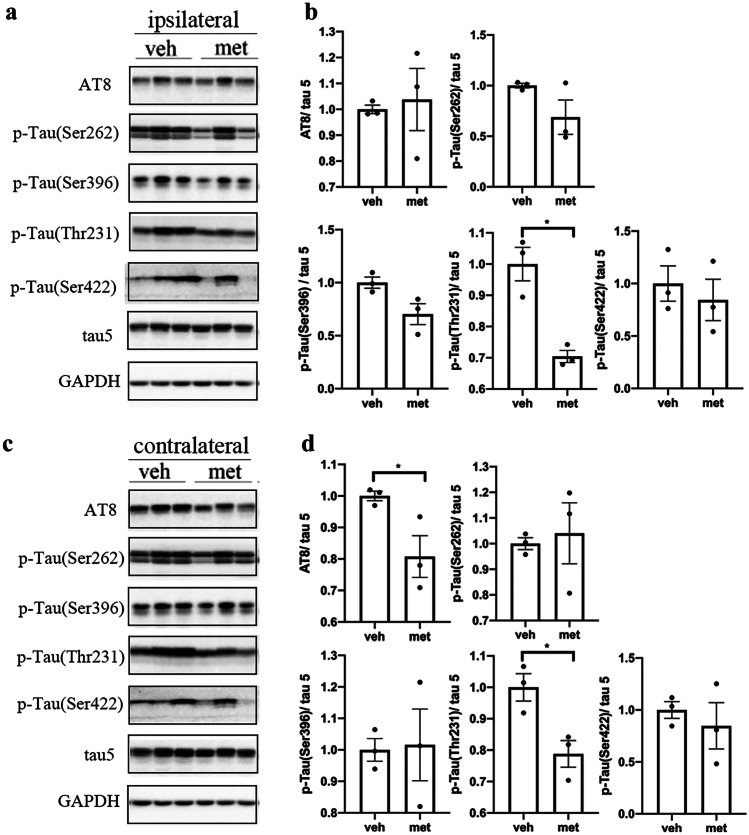
Metformin reduced the phosphorylation of tau in the cortex of tau-seeded PS19 mice. Representative Western blots of brain extracts from the ipsilateral (**a**) and contralateral (**c**) cortices in tau-seeded PS19 mice developed with phosphorylation-dependent and site-specific tau antibodies and tau5 against total tau. Densitometric quantification of blots after normalization to tau5 levels (**b**, **d**). The data were analyzed by Student’s *t*-test. Values are presented as mean ± SEM. *P < 0.05 vs. veh group. *n* = 3 per group. Veh, vehicle; met, metformin

**Fig. 4:**
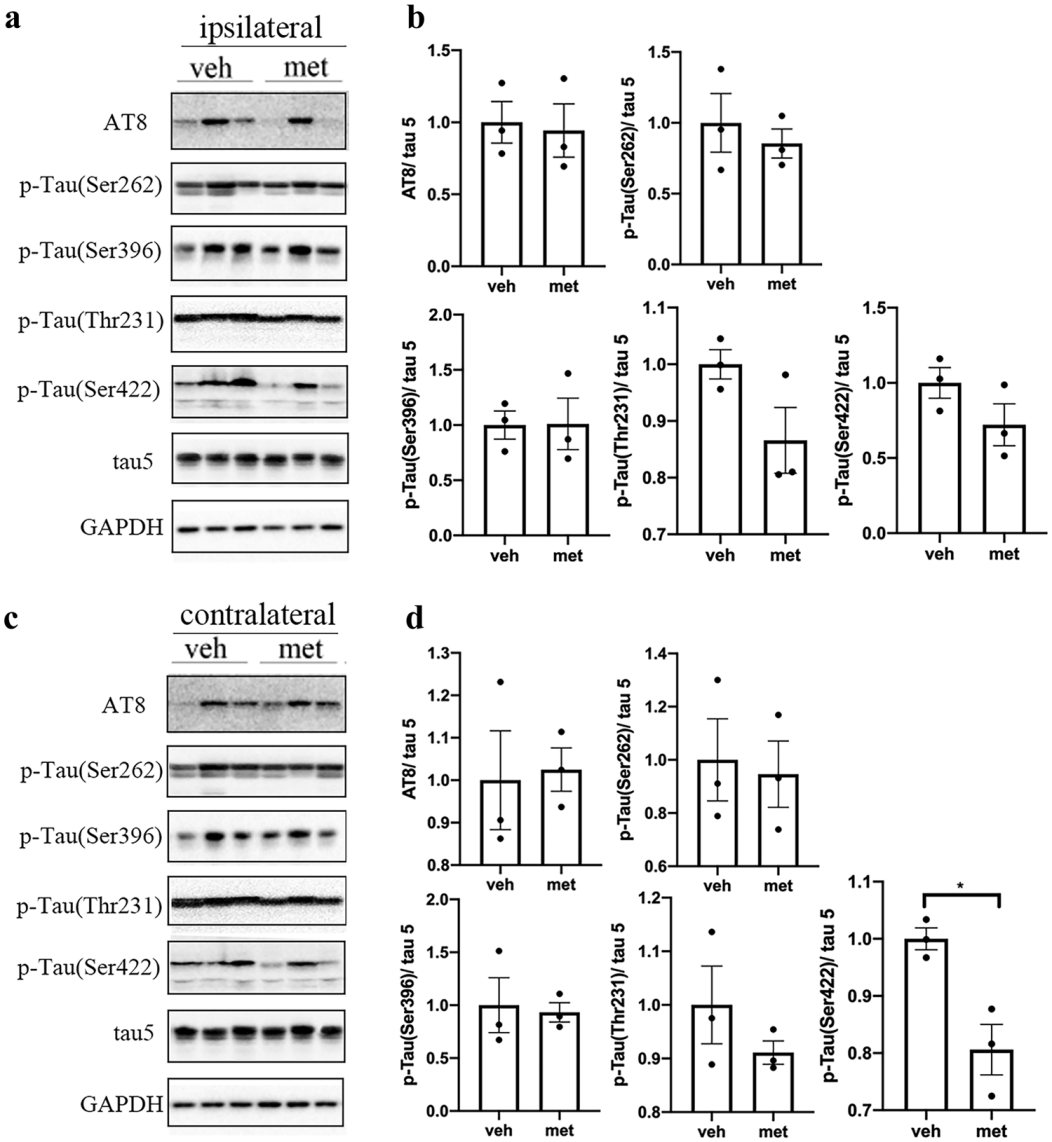
Metformin reduced the phosphorylation of tau in the hippocampus of tau-seeded PS19 mice. Representative Western blots of brain extracts from the ipsilateral (**a**) and contralateral (**c**) hippocampi in tau-seeded PS19 mice developed with phosphorylation-dependent and site-specific tau antibodies and tau5 against total tau. Densitometric quantification of blots after normalization to tau5 levels (**b**, **d**). The data were analyzed by Student’s *t-*test. Values are presented as mean ± SEM. *P < 0.05 vs. veh group. *n* = 3 per group. Veh, vehicle; met, metformin

It has been demonstrated that tau phosphorylation plays an important role in the spread of tau pathology [27]. We examined tau phosphorylation at multiple sites in the brains of PS19 mice to investigate the effects of metformin on tau phosphorylation in tau spreading. It was found that metformin decreased the levels of tau phosphorylation at Thr231 but not at Ser202/Thr205 (AT8), Ser262, Ser396, or Ser422 in the ipsilateral cortex of tau-seeded PS19 mice (Fig. 3a, b). Tau phosphorylation levels were also reduced in the contralateral cortex of tau-seeded PS19 mice treated with metformin at Ser202/Thr205 and Thr231 (Fig. 3c, d). Moreover, metformin treatment reduced the tau phosphorylation only at Ser422 in the contralateral hippocampus of tau-seeded PS19 mice (Fig. 4). These findings indicate that metformin slightly reduced the levels of tau phosphorylation in the brains of tau-seeded PS19 mice.

**Fig. 5:**
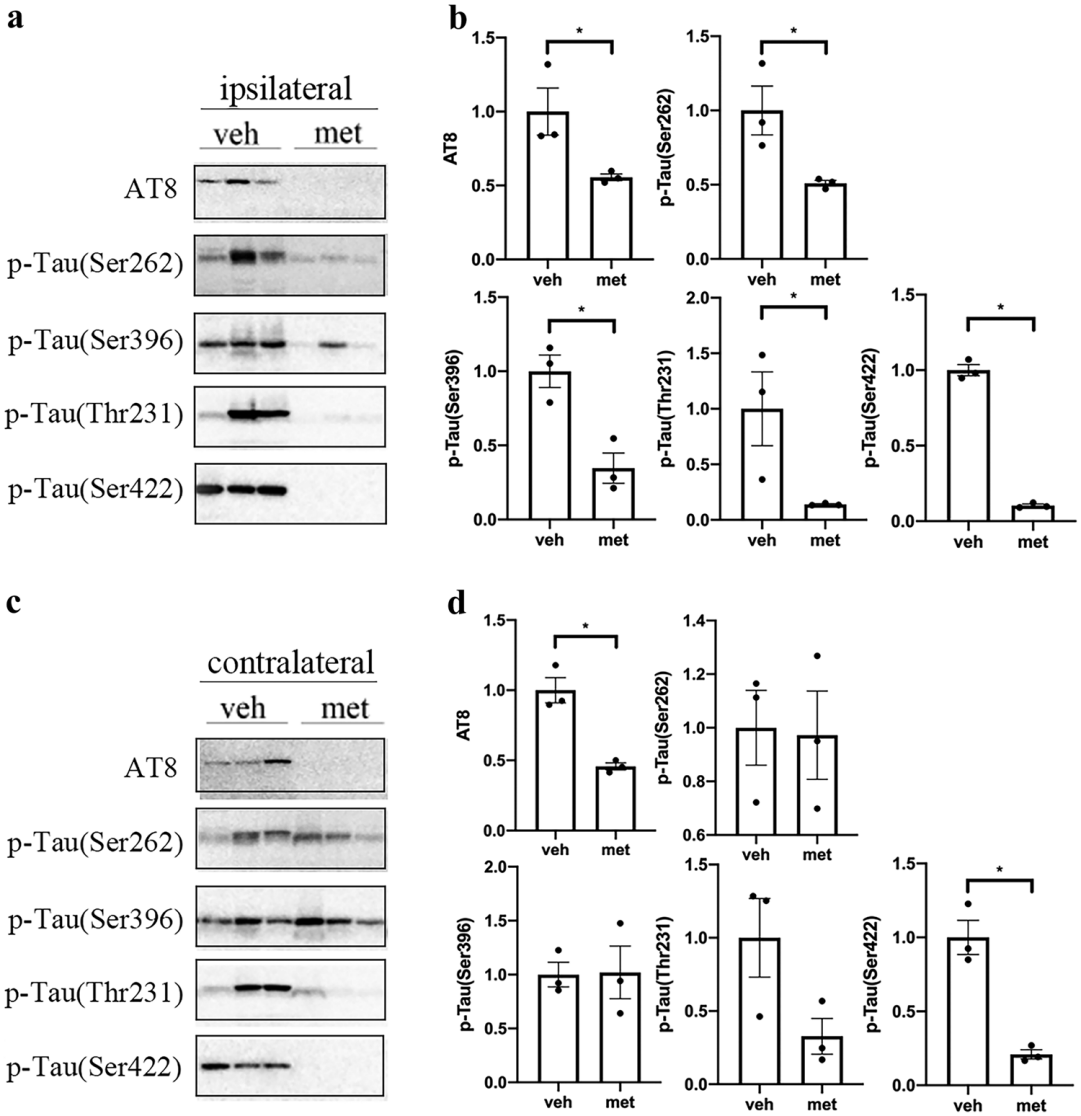
Metformin reduced the hyperphosphorylation of insoluble tau in the cortex of tau-seeded PS19 mice. Representative Western blots of the sarkosyl insoluble fraction of brain exacts from the ipsilateral (**a**) and contralateral (**c**) cortices of the tau-seeded PS19 developed with phosphorylation-dependent and site-specific tau antibodies. Densitometric quantification of blots (**b**, **d**). The data were analyzed by Student’s *t*-test. Values are presented as mean ± SEM. *P < 0.05 vs. veh group. *n* = 3 per group. Veh, vehicle; met, metformin

**Fig. 6:**
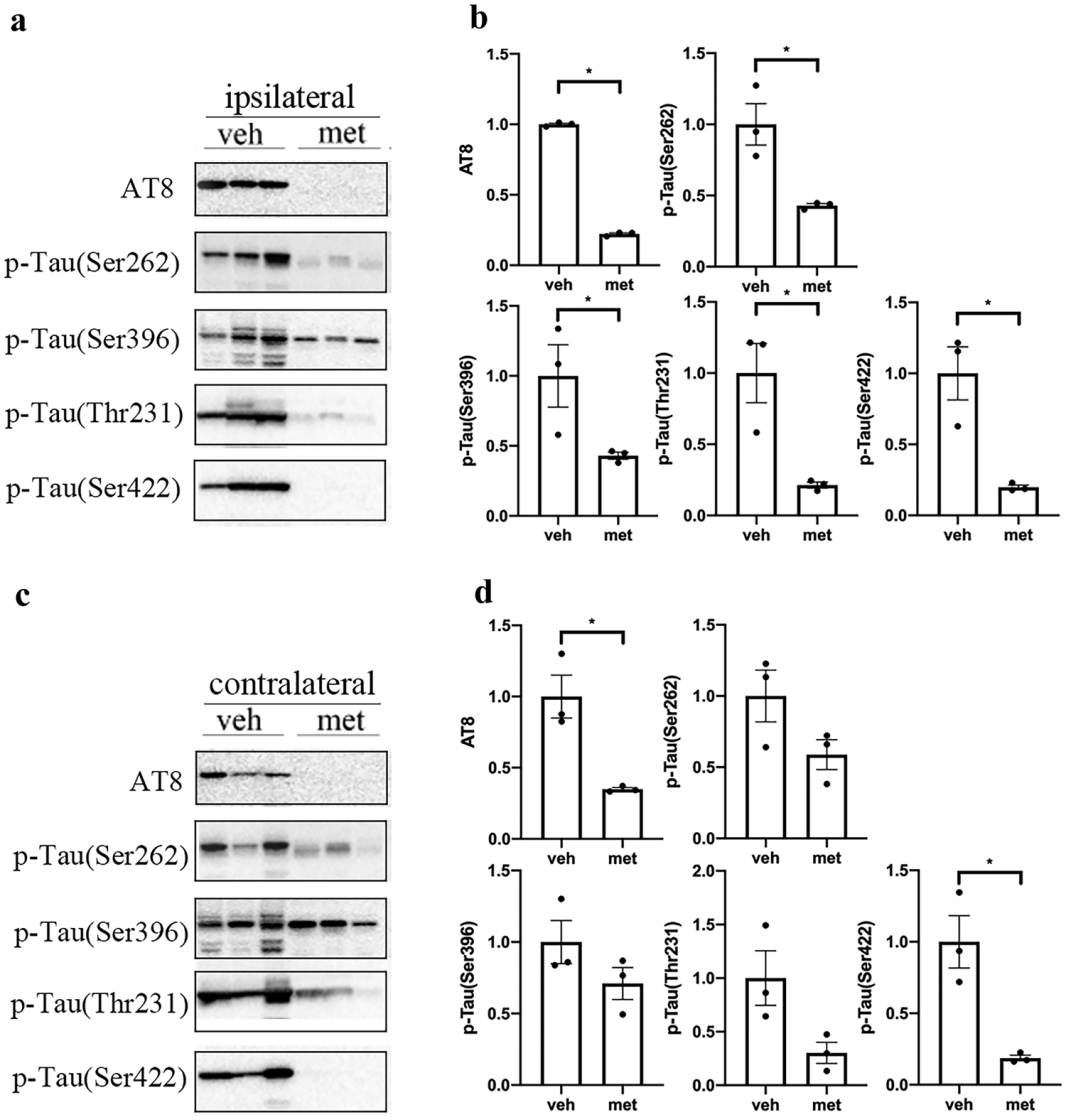
Metformin reduced the hyperphosphorylation of insoluble tau in the hippocampus of tau-seeded PS19 mice. Representative Western blots of the sarkosyl insoluble fraction of brain exacts from the ipsilateral (**a**) and contralateral (**c**) hippocampi of the tau-seeded PS19 mice developed with phosphorylation-dependent and site-specific tau antibodies. Densitometric quantification of blots (**b**,** d**). The data were analyzed by Student’s *t*-test. Values are presented as mean ± SEM. *P < 0.05 vs. veh group. *n* = 3 per group. Veh, vehicle; met, metformin

Furthermore, we investigate the effect of metformin on the phosphorylation of insoluble tau fractions in the brains of tau-seeded PS19 mice. The tau phosphorylation at Ser202/Thr205 (AT8), Ser262, Ser396, Thr231, and Ser422 was significantly reduced in the ipsilateral cortex and hippocampus of tau-seeded PS19 mice treated with metformin compared to the mice treated with normal drinking water (Figs. 5a, b and 6a, b). However, tau phosphorylation was significantly reduced at Ser202/Thr205 (AT8) and Ser422 but not at Ser262, Ser396, or Thr231 in the contralateral cortex and hippocampus of metformin-treated mice (Figs. 5c, d and 6c, d). These findings revealed that metformin significantly reduced tau phosphorylation in the insoluble fraction of tau in tau-seeded PS19 mice.

### Metformin Had No Effects on Protein Levels of Tau Kinases or Phosphatases but Reduced Protein Levels of mTORC1

**Fig. 7:**
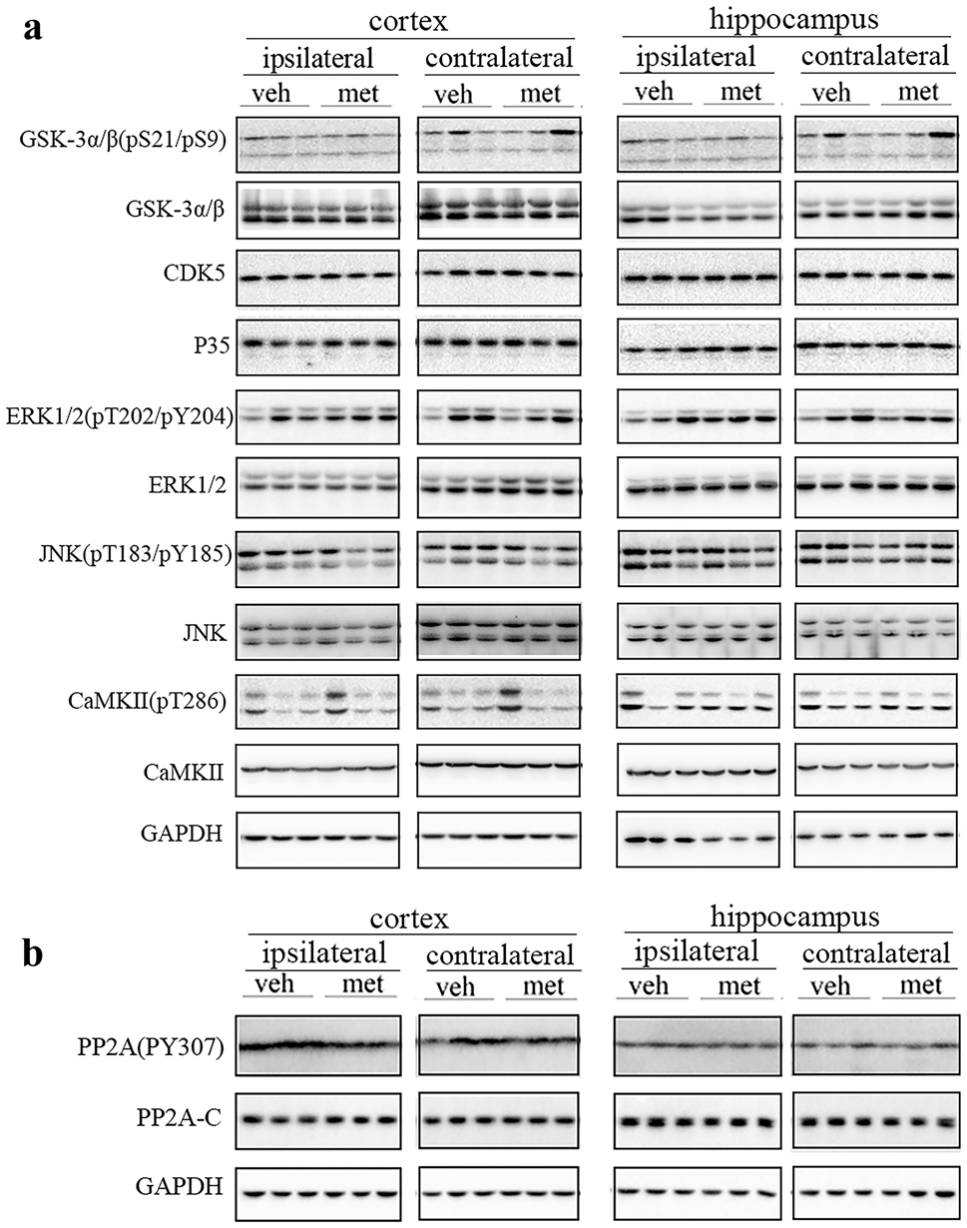
Metformin had no effects on protein levels of tau kinases or tau phosphatases. Representative Western blots of tau kinases (**a**) and PP2A (**b**) in the cortices and hippocampi of tau-seeded PS19 mice. *n* = 3 per group. Veh, vehicle; met, metformin

It has been widely recognized that tau hyperphosphorylation is closely related to tau kinases and phosphatase activity. The levels of tau kinases and phosphatase and their activity-related phosphorylation were determined to investigate the mechanisms underlying the reduced levels of tau phosphorylation. The major tau kinases involved in AD are glycogen synthase kinase-3α/β (GSK-3α/β), GSK-3α/β (pS21/pS9), cyclin-dependent kinase 5 (CDK5) and its activator P35, extracellular signal-regulated kinase (ERK), ERKl/2(pT202/pY204), c-Jun N-terminal kinase (JNK), JNK (pT183/pY185), calcium/calmodulin-dependent protein kinase II (CaMKII), and CaMKII (pT286). However, no differences in these tau kinase levels were found between metformin and vehicle-treated PS19 mice (Fig. 7a). These results indicate that tau kinases might not be involved in the reduced tau phosphorylation associated with metformin treatment.

**Fig. 8:**
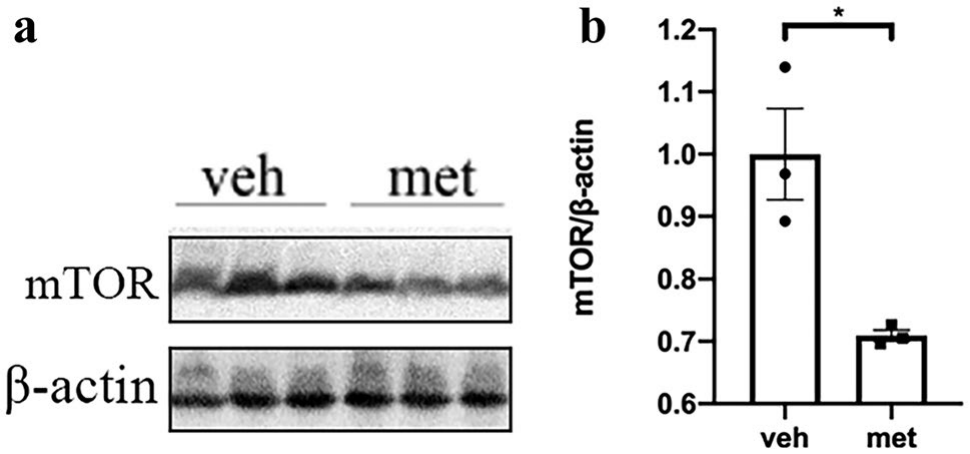
Metformin reduced protein levels of mTORC1. **a** Representative Western blots of mTORC1 in the brains of tau-seeded PS19 mice. **b** Densitometric quantification of blots after normalization to β-actin levels. The data were analyzed by Student’s *t*-test. Values are presented as mean ± SEM. *P < 0.05 vs. veh group. *n* = 3 per group. Veh, vehicle; met, metformin

Protein phosphatase 2A (PP2A) is the major tau phosphatase, and its activity is significantly reduced in the brain of AD patients. Phosphorylation of PP2A catalytic subunit (PP2A-C) at Tyr307 (PP2A(PY307)) inhibits PP2A activity. We found no differences in PP2A-C or PP2A(pY307) levels between the metformin and vehicle-treated PS19 mice (Fig. 7b), indicating that metformin had no effects on the PP2A protein levels. Numerous studies have shown that mTOR can negatively regulate PP2A activity [28, 29]. mTORC1 protein levels were lower in the brains of tau-seeded PS19 mice treated with metformin (Fig. 8).

### Metformin Attenuated Learning and Memory Deficits in Tau-Seeded PS19 Mice

**Fig. 9:**
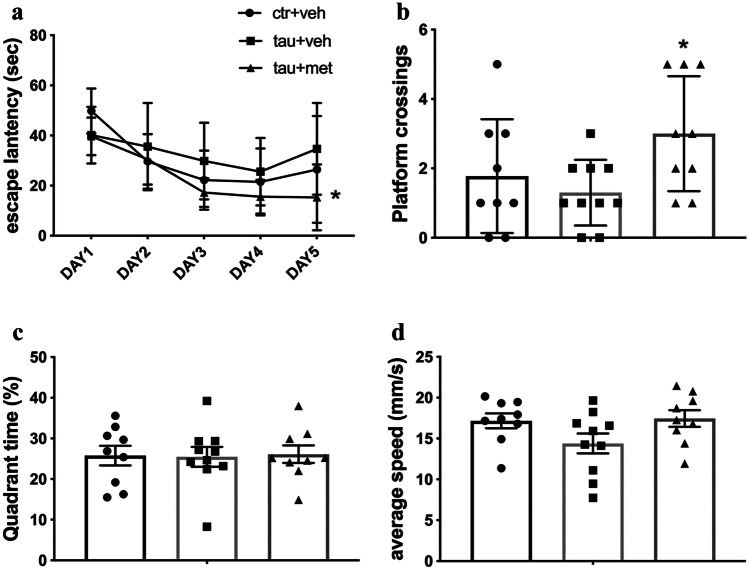
Metformin attenuated learning and memory deficits in tau-seeded PS19 mice. In the acquisition phase, escape latencies (**a**) and average swim speed (**d**) were recorded. In the probe trial, the number of platform crossings (**b**) and the time in the target quadrant (**c**) were recorded. For comparisons of escape latency, a two-way repeated-measures ANOVA was utilized with Fisher’s LSD post hoc tests. For other data, a one-way ANOVA followed by Bonferroni post hoc test was used. The data are expressed as the means ± SEM. Ctr + veh, ^WT^BE-injected PS19 mice treated with normal drinking water; tau + veh, ^PS19^BE-injected PS19 mice treated with normal drinking water; tau + met, ^PS19^BE-injected PS19 mice treated with metformin. *P < 0.05 vs. tau + veh group. *n* = 9–10 per group

The MWM was used to investigate the effects of metformin on the cognition of PS19 mice. The reduced latency to find the platform demonstrated that all mice learned to locate the hidden platform during the 4-day acquisition phase (Fig. 9a). Metformin significantly reduced the escape latency of tau-seeded PS19 mice, indicating that metformin improved spatial learning of these mice (Fig. 9a). Furthermore, tau-seeded PS19 mice treated with metformin had more platform crossings in the probe trial, indicating that these mice have better spatial memory (Fig. 9b). Differences in mouse motor ability did not cause the observed differences, as evident by the lack of any significant difference in swimming speed of mice between groups (Fig. 9d). There were no differences in time spent in the former platform quadrant were (Fig. 9c). The results indicate that metformin improved spatial learning and memory deficits in tau-seeded PS19 mice.

## Discussion

In the current study, we explored the effect of metformin on tau spreading in the brains of PS19 mice. Tau spreading in PS19 mice brains was induced by unilateral injection of tau seeds into the hippocampus and the overlying cortex. Metformin treatment for four months reduced tau phosphorylation, particularly in the insoluble fraction. Moreover, metformin attenuated the spreading of tau pathology in the ipsilateral and reduced tau pathology in the contralateral hemispheres. Metformin also improved the spatial learning and memory ability of tau-seeded PS19 mice.

In the present study, metformin significantly reduced tau phosphorylation at multiple sites, particularly in the insoluble fraction. Tau hyperphosphorylation plays a decisive role in the spread of tau pathology [27]. It was found that the injection of tau seeds containing pathological tau protein extracted from AD patients (AD P-tau) into the brains of mice induced widespread tau pathology. However, injection of dephosphorylated AD P-tau failed to induce tau spreading, demonstrating that the tau species that can be taken up by neurons and spread among neurons is primarily phosphorylated tau [27, 30]. Importantly, tau phosphorylation was only observed at Ser422 in the contralateral hippocampus of AD P-tau-seeded Tg/hTau mice, implying that Ser422 phosphorylation may be an early event in AD P-tau-induced tau hyperphosphorylation and occurs in the early stage of tau spreading [31]. We found that metformin reduced tau phosphorylation at Ser422 in the contralateral hippocampus of tau-seeded PS19 mice, particularly in the insoluble tau fraction, implying that metformin may inhibit tau phosphorylation in the initial period and thus effectively attenuate tau propagation. The main component of NFTs is sarkosyl insoluble tau. Metformin significantly reduced tau phosphorylation at Ser202/Thr205 in the sarkosyl insoluble fraction, which is consistent with the decreased density of AT8-positive neurons in the bilateral cortex and hippocampus. This further indicates the lower abundance of NFTs in metformin-treated mice.

It is well known that hyperphosphorylated tau seeds capture template tau aggregation, eventually spreading tau pathology [32]. Sarkosyl-insoluble tau is more resistant to dephosphorylation than soluble tau, resulting in tau hyperphosphorylation [31]. Metformin treatment significantly reduced the phosphorylation of sarkosyl insoluble tau and decreased tau aggregates. In this context, we hypothesize that metformin inhibited tau spreading by reducing phosphorylation of sarkosyl insoluble tau, abolished the ability of tau seeds to template normal tau proteins, and prevented tangle formation.

Tau phosphorylation is closely related to tau kinases and phosphatase PP2A. However, changes in the total or phosphorylated levels of tau kinases and phosphatase were not observed. We were unable to measure the activity of the kinases and phosphatase. Therefore, we investigated the level of mTORC1, an upstream factor of PP2A that has been shown to negatively regulate the activity of PP2A [28, 29]. We found that metformin significantly reduced mTORC1 levels. Metformin may inhibit mTORC1 while activating PP2A, resulting in tau protein dephosphorylation. Further research involving the investigation of PP2A activity will provide additional information about the mechanisms underlying the role of metformin in tau phosphorylation.

In the current study, we found decreased tau phosphorylation in the brain homogenates of tau-seeded PS19 mice treated with metformin and attenuated tau pathology in tau-seeded mice with AT8 staining. Reduced density of AT8 staining in the ipsilateral hemisphere of tau-seeded PS19 mice suggests reduced seeding induction of tau pathology by metformin. However, the lower AT8 density in the contralateral hemisphere could also be due to decreased efficiency of tau seeding induction in the injected hemisphere. Therefore, metformin may have reduced tau propagation to the contralateral hemisphere either directly or indirectly.

We previously observed seeded NP tau aggregates in ^PS19^BE-injected APP/PS1 mice, and metformin administration reduced NP tau spreading [25], which is consistent with the current findings. Altogether, these findings confirm that metformin may attenuate the spreading of tau pathology. Interestingly, a previous study found that metformin promoted tau protein dephosphorylation but accelerated tau pathology aggregation in the brains of P301S mice [23]. This could be due to different concurring mechanisms. They found that metformin has pro-aggregation effects in vitro, which result in the formation of tau aggregates through direct effects. These tau aggregates, however, may not have the propagation property. In contrast, when recombinant tauP301S aggregates were incubated with both heparin and metformin in vitro, the fluorescence value was reduced compared to that of heparin alone [23]. These findings demonstrated that metformin interfered with the process of heparin-induced aggregation in vitro. Furthermore, metformin experiments on recombinant tauP301S in vitro may not accurately mimic the effects of tau dephosphorylation in vivo. The present study focuses on the effects of metformin on tau spreading, a process in which tau hyperphosphorylation plays a crucial role. We hypothesize that the tau dephosphorylation by metformin outweighs its pro-aggregation property. Therefore, the difference in results may be due to the different animal models.

Epidemiological studies have shown that metformin treatment significantly reduced the risk of cognitive deficits in older adults with T2DM in longitudinal and cross-sectional analyses [17–19]. Metformin administration for 12 months is associated with better performance in selective reminding tests in overweight patients aged 55 to 90 with amnestic mild cognitive impairment [33]. However, a negative association of metformin with cognition has also been reported. Long-term metformin treatment increased the risk of AD in T2DM patients over 60, but there was no consistent trend with the increasing metformin dose, indicating the involvement of other confounding factors [34]. Notably, it has been reported that the negative effect of metformin on cognition may be associated with metformin-related vitamin B_12_ deficiency [35]. In contrast, it has been proposed that the effects of metformin on cognition performance may vary depending on the risk profile of the patients [36].

Based on the evidence, metformin has some potential for AD disease modification. However, many clinical trials and basic research are still required to demonstrate the therapeutic value of metformin on AD. In the present study, we found that metformin reduced tau hyperphosphorylation, ameliorated tau spreading pathology, and improved the learning and memory impairment in tau-seeded PS19 mice. In a previous study, we also found reduced Aβ deposits and NP tau spreading in tau-seeded APP/PS1 mice, which could be attributed to increased microglial phagocytosis [25]. Overall, our studies suggest a beneficial role of metformin in limiting the propagation of tau pathology, which could be the underlying mechanism for the decreased AD risk for metformin users.

## Conclusions

In conclusion, metformin reduced tau phosphorylation, attenuated tau pathology in tau-seeded mice, and improved learning and memory impairment. These findings provide experimental evidence supporting the potential role of metformin in the early prevention/treatment of AD. In the early stages of AD, metformin intervention may inhibit or even block the transmission of tau pathology, slowing disease progression.

## Supplementary Information

Below is the link to the electronic supplementary material.

Supplementary file1 (DOCX 1305 kb)

Supplementary file2 (DOCX 33 kb)

Supplementary file3 (DOCX 33 kb)

Supplementary file4 (DOCX 33 kb)

Supplementary file5 (DOCX 33 kb)

Supplementary file6 (DOCX 33 kb)

Supplementary file7 (DOCX 33 kb)

Supplementary file8 (DOCX 33 kb)

Supplementary file9 (DOCX 33 kb)

Supplementary file10 (DOCX 33 kb)

Supplementary file11 (DOCX 33 kb)

Supplementary file12 (DOCX 33 kb)

Supplementary file13 (DOCX 33 kb)

Supplementary file14 (DOCX 33 kb)

## References

[1] Huang Y, Mucke L (2012). Alzheimer mechanisms and therapeutic strategies. Cell.

[2] Korczyn AD (2008). The amyloid cascade hypothesis. Alzheimers Dement.

[3] Braak H, Braak E (1995). Staging of Alzheimer’s disease-related neurofibrillary changes. Neurobiol Aging.

[4] Braak H, Braak E (1991). Neuropathological stageing of Alzheimer-related changes. Acta Neuropathol.

[5] Frost B, Diamond MI (2010). Prion-like mechanisms in neurodegenerative diseases. Nat Rev Neurosci.

[6] Walker LC, Diamond MI, Duff KE, Hyman BT (2013). Mechanisms of protein seeding in neurodegenerative diseases. JAMA Neurol.

[7] Clavaguera F, Tolnay M, Goedert M (2017). The prion-like behavior of assembled tau in transgenic mice. Cold Spring Harb Perspect Med.

[8] de Calignon A, Polydoro M, Suarez-Calvet M, William C, Adamowicz DH, Kopeikina KJ (2012). Propagation of tau pathology in a model of early Alzheimer’s disease. Neuron.

[9] Ahmed Z, Cooper J, Murray TK, Garn K, McNaughton E, Clarke H (2014). A novel in vivo model of tau propagation with rapid and progressive neurofibrillary tangle pathology: the pattern of spread is determined by connectivity, not proximity. Acta Neuropathol.

[10] Clavaguera F, Bolmont T, Crowther RA, Abramowski D, Frank S, Probst A (2009). Transmission and spreading of tauopathy in transgenic mouse brain. Nat Cell Biol.

[11] Iba M, Guo JL, McBride JD, Zhang B, Trojanowski JQ, Lee VM (2013). Synthetic tau fibrils mediate transmission of neurofibrillary tangles in a transgenic mouse model of Alzheimer’s-like tauopathy. J Neurosci.

[12] Zhang J, Chen C, Hua S, Liao H, Wang M, Xiong Y (2017). An updated meta-analysis of cohort studies: diabetes and risk of Alzheimer’s disease. Diabetes Res Clin Pract.

[13] Banks WA, Owen JB, Erickson MA (2012). Insulin in the brain: there and back again. Pharmacol Ther.

[14] Ascher-Svanum H, Chen YF, Hake A, Kahle-Wrobleski K, Schuster D, Kendall D (2015). Cognitive and functional decline in patients with mild Alzheimer dementia with or without comorbid diabetes. Clin Ther.

[15] Steen E, Terry BM, Rivera EJ, Cannon JL, Neely TR, Tavares R (2005). Impaired insulin and insulin-like growth factor expression and signaling mechanisms in Alzheimer’s disease–is this type 3 diabetes?. J Alzheimers Dis.

[16] Rotermund C, Machetanz G, Fitzgerald JC (2018). The therapeutic potential of metformin in neurodegenerative diseases. Front Endocrinol (Lausanne).

[17] Hsu CC, Wahlqvist ML, Lee MS, Tsai HN (2011). Incidence of dementia is increased in type 2 diabetes and reduced by the use of sulfonylureas and metformin. J Alzheimers Dis.

[18] Cheng C, Lin CH, Tsai YW, Tsai CJ, Chou PH, Lan TH (2014). Type 2 diabetes and antidiabetic medications in relation to dementia diagnosis. J Gerontol A Biol Sci Med Sci.

[19] Orkaby AR, Cho K, Cormack J, Gagnon DR, Driver JA (2017). Metformin vs sulfonylurea use and risk of dementia in US veterans aged >/=65 years with diabetes. Neurology.

[20] Allard JS, Perez EJ, Fukui K, Carpenter P, Ingram DK, de Cabo R (2016). Prolonged metformin treatment leads to reduced transcription of Nrf2 and neurotrophic factors without cognitive impairment in older C57BL/6J mice. Behav Brain Res.

[21] Chen F, Dong RR, Zhong KL, Ghosh A, Tang SS, Long Y (2016). Antidiabetic drugs restore abnormal transport of amyloid-beta across the blood-brain barrier and memory impairment in db/db mice. Neuropharmacology.

[22] Pintana H, Apaijai N, Pratchayasakul W, Chattipakorn N, Chattipakorn SC (2012). Effects of metformin on learning and memory behaviors and brain mitochondrial functions in high fat diet induced insulin resistant rats. Life Sci.

[23] Barini E, Antico O, Zhao Y, Asta F, Tucci V, Catelani T (2016). Metformin promotes tau aggregation and exacerbates abnormal behavior in a mouse model of tauopathy. Mol Neurodegener.

[24] Chen JL, Luo C, Pu D, Zhang GQ, Zhao YX, Sun Y (2019). Metformin attenuates diabetes-induced tau hyperphosphorylation in vitro and in vivo by enhancing autophagic clearance. Exp Neurol.

[25] Chen Y, Zhao S, Fan Z, Li Z, Zhu Y, Shen T (2021). Metformin attenuates plaque-associated tau pathology and reduces amyloid-beta burden in APP/PS1 mice. Alzheimers Res Ther.

[26] He Z, Guo JL, McBride JD, Narasimhan S, Kim H, Changolkar L (2018). Amyloid-beta plaques enhance Alzheimer’s brain tau-seeded pathologies by facilitating neuritic plaque tau aggregation. Nat Med.

[27] Hu W, Zhang X, Tung YC, Xie S, Liu F, Iqbal K (2016). Hyperphosphorylation determines both the spread and the morphology of tau pathology. Alzheimers Dement.

[28] Wlodarchak N, Xing Y (2016). PP2A as a master regulator of the cell cycle. Crit Rev Biochem Mol Biol.

[29] Kickstein E, Krauss S, Thornhill P, Rutschow D, Zeller R, Sharkey J (2010). Biguanide metformin acts on tau phosphorylation via mTOR/protein phosphatase 2A (PP2A) signaling. Proc Natl Acad Sci U S A.

[30] Takeda S, Wegmann S, Cho H, DeVos SL, Commins C, Roe AD (2015). Neuronal uptake and propagation of a rare phosphorylated high-molecular-weight tau derived from Alzheimer’s disease brain. Nat Commun.

[31] Miao J, Shi R, Li L, Chen F, Zhou Y, Tung YC (2019). Pathological tau from Alzheimer’s brain induces site-specific hyperphosphorylation and SDS- and reducing agent-resistant aggregation of tau in vivo. Front Aging Neurosci.

[32] D'Errico P, Meyer-Luehmann M (2020). Mechanisms of pathogenic tau and Aβ protein spreading in Alzheimer’s disease. Front Aging Neurosci..

[33] Luchsinger JA, Perez T, Chang H, Mehta P, Steffener J, Pradabhan G (2016). Metformin in amnestic mild cognitive impairment: results of a pilot randomized placebo controlled clinical trial. J Alzheimers Dis.

[34] Imfeld P, Bodmer M, Jick SS, Meier CR (2012). Metformin, other antidiabetic drugs, and risk of Alzheimer’s disease: a population-based case-control study. J Am Geriatr Soc.

[35] Chapman LE, Darling AL, Brown JE (2016). Association between metformin and vitamin B12 deficiency in patients with type 2 diabetes: a systematic review and meta-analysis. Diabetes Metab.

[36] Wang C-P, Lorenzo C, Habib SL, Jo B, Espinoza SE (2017). Differential effects of metformin on age related comorbidities in older men with type 2 diabetes. J Diabetes Complications.

