# Supplementary material for: Metformin Attenuates Tau Pathology in Tau-Seeded PS19 Mice
Source: Neurotherapeutics. 2022 Nov 23;20(2):452–63. doi: 10.1007/s13311-022-01316-6 (PMC10121992; doi:10.1007/s13311-022-01316-6)
Supplement: Supplementary file 1 — Supplementary file1 (DOCX 1305 kb) [file 13311_2022_1316_MOESM1_ESM.docx]

**METHODS**

**Dot blot**

Tau level of the injected brain homogenates was assayed using dot blots, as previously described^1^. Briefly, samples of different concentrations were applied onto a nitrocellulose (NC) membrane (Millipore, Bedford, MA, USA), which was then placed in a 37 ℃ oven for 1 h to allow the protein to bind to the membrane. Next, membranes were blocked in 5% BSA in 0.1% TBST for 1 h and then incubated overnight with 4Rtau antibody at 4 ℃. The next day, membranes were incubated with the appropriate secondary antibody for 1 h at room temperature. Positive antibody binding was then visualized using ECL-PLUS system with ECL. Signal intensity was analyzed with Image Lab.

**Negative staining transmission** **electron microscopy**

Negative staining transmission electron microscopy was performed, as previously described^2^. Briefly, brain extract was placed on 300 meshed carbon-coated copper grids, stained with one drop of 2% uranyl acetate and visualized with Tecnai G2 spirit transmission electron microscope (Thermo FEI, CZ).

**FIGURE LEGENDS**


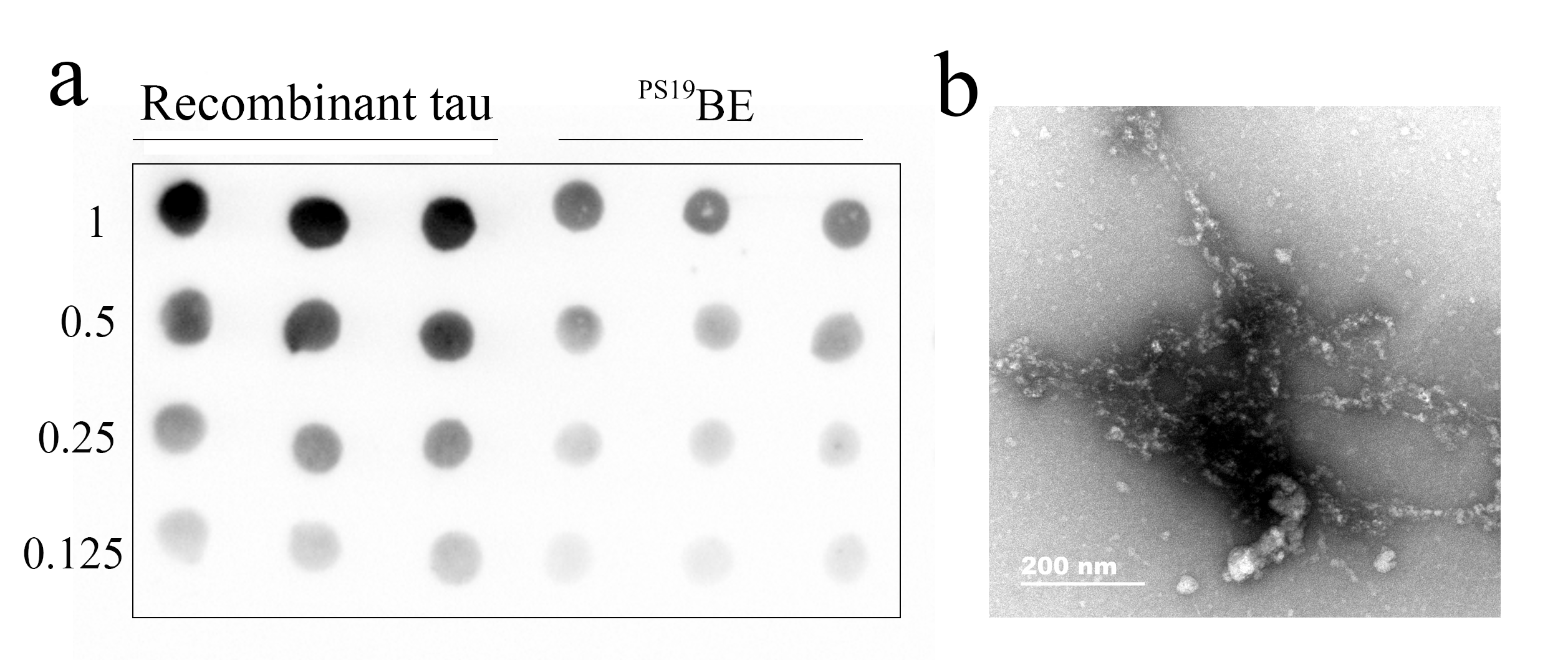


**Sup. Fig. 1. Experiment schedule** **of the study.**


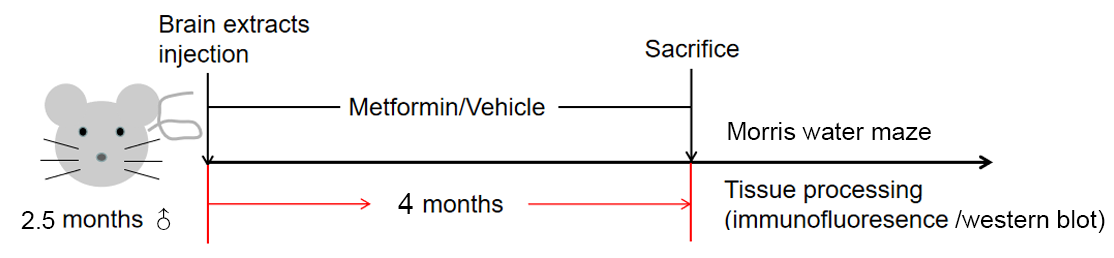


**Sup. Fig. 2. Characterization of injected brain extracts. (a)** Dot blots of injected brain homogenates. Recombinant tau was used to generate a standard curve, and tau concentration of brain extracts was calculated as 61.82 µg/mL. (**b)** Representative image of brain extracts obtained by negative staining transmission electron microscope.

**REFERENCES**

1. Li L, Shi R, Gu J, et al. Alzheimer's disease brain contains tau fractions with differential prion-like activities. Acta Neuropathol Commun. 2021;9(1):28.

2. Barini E, Antico O, Zhao Y, et al. Metformin promotes tau aggregation and exacerbates abnormal behavior in a mouse model of tauopathy. Mol Neurodegener. 2016;11:16.
